# Invasive sphenoid sinus aspergillosis mimicking sellar tumor: a report of 4 cases and systematic literature review

**DOI:** 10.1186/s41016-020-00187-0

**Published:** 2020-04-09

**Authors:** Hanwen Zhang, Nian Jiang, Xuelei Lin, Siyi Wanggou, Jeffrey J. Olson, Xuejun Li

**Affiliations:** 1 Department of Neurosurgery, Xiangya Hospital, Central South University, 87 Xiangya Road, Changsha, 410008 Hunan People’s Republic of China; 2 Department of Neurosurgery, Emory University School of Medicine, Atlanta, GA 30322 USA

**Keywords:** Invasive fungal sinusitis, Cavernous sinus syndrome, Intracranial aspergillosis, Sphenoid sinus infection, Sellar mass, Imaging features, Prognosis

## Abstract

**Background:**

Invasive sphenoid sinus aspergillosis is a rare but life-threatening condition usually found in immunocompromised patients. When involving cavernous sinus and surrounding structures, patients are frequently misdiagnosed with a neoplasm or sellar abscess. Timely diagnosis and intervention are crucial to patients’ outcomes. The objective of this study is to review cases of invasive sphenoid sinus aspergillosis to describe disease manifestations, imaging features, treatment, and outcome.

**Case presentation:**

We describe four patients with invasive sphenoid sinus aspergillosis misdiagnosed as sellar tumors preoperatively. The mass was completely removed in three patients and partially removed in one patient microscopically. Pathological examinations confirmed *Aspergillus* in all cases. All four patients received anti-fungal agents postoperatively. There was no recurrence at the time of each patient’s follow-up date. One patient with complete resection was lost to follow-up while the other three patients’ neurologic function improved. Additionally, we performed a systematic review regarding invasive sphenoid sinus aspergillosis of existing English literature.

**Conclusion:**

With regard to clinical symptoms, headache, vision impairment, and ophthalmoplegia were observed in over half of the patients in the literature. A sellar mass with bone destruction on CT and involvement of cavernous sinus is highly suggestive of invasive fungal sphenoid sinusitis. Immediate surgical removal of the lesion is recommended for invasive sphenoid sinus aspergillosis to preserve nerve function and increase the likelihood of survival.

## Background

Intracranial aspergillosis is an extremely rare but life-threatening disease. *Aspergillus* can reach the central nervous system (CNS) by three different routes [1]. When it directly spreads from sphenoid sinus to cavernous sinus, patients are frequently misdiagnosed with a neoplasm or sellar abscess due to the lack of specificity on radiological images. Without proper intervention, the mortality rate can be as high as 80% [2]. It is imperative to realize that accurate diagnosis is crucial for patients to achieve a better prognosis [3]. The current treatment of intracranial aspergillosis usually comprises a combination of surgical debridement and antifungal medication [4, 5]. However, a retrospective evaluation and summary of the outcomes reported in the literature is needed to guide further clinical intervention. The aim of this study is to analyze the radiological features and treatment options so as to evaluate their effect on outcomes of invasive sphenoid sinus aspergillosis.

## Case presentation

### Case 1

**Fig. 1:**
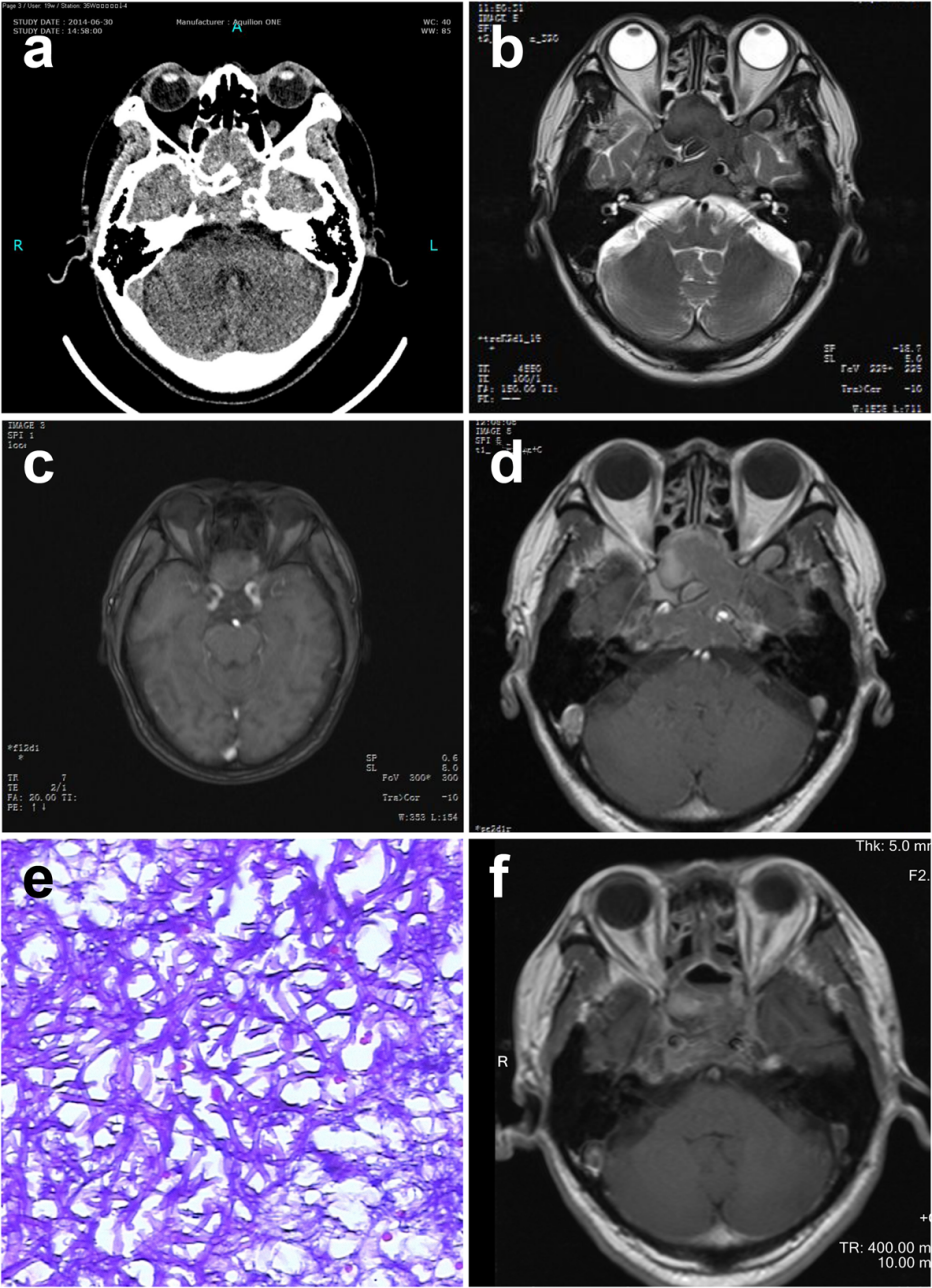
Case 1: a 69-year-old man. **a** CT image showing bone destruction in the sphenoid sinus and clival region. **b** MRI showing an intrasellar mass with hypointense signal on T2WI. **c** MRI showing an intrasellar mass with isointense signal on T1WI. **d** Enhanced MRI showing the lesions with heterogeneous enhancement in sphenoid, bilateral cavernous sinuses, ethmoid sinuses, and maxillary sinus. **e** Photomicrograph of the surgical specimen revealing the histologic findings of septate, clustered aspergillus hyphae. **f** MRI showing the resection of the mass

A 69-year-old male was admitted with a progressive frontal paroxysmal headache accompanied with mild vomiting, diplopia, and visual disturbance for 1 month, without fever. Visual acuity was 0.6 in the left eye and 0.9 in the right eye without visual field loss. Other neurologic and clinical findings were normal. He was healthy other than a 10-year history of well-controlled hypertension and coronary heart disease. The patient reported no history of fungal infection or a history of contact with unusual infectious sources. CT scan showed a mass in the sphenoid sinus and clival region. Bone destruction was observed in both regions (Fig. 1a). MRI showed a hypointense signal on T2-weighted images (T2WI) in the lesions and an isointense signal on T1 weighted images (T2WI) with heterogeneous gadolinium-enhancement in the sphenoid sinus, both cavernous sinuses, bilateral ethmoid sinuses, and maxillary sinus (Fig. 1b–d). This patient underwent trans-sphenoidal lesion debridement. Purulence was observed during the operation. A biopsy was performed immediately and histopathology examination revealed *Aspergillus* species (Fig. 1e). The patient was administered fluconazole after surgery. Post-operative neurological examination showed obvious improvement of headache, vomiting, vision, and diplopia. Post-operative images indicated the resection of the mass (Fig. 1f). Examination after a follow-up period of 24 months showed no clinical symptoms, and no additional therapy was required.

### Case 2

**Fig. 2:**
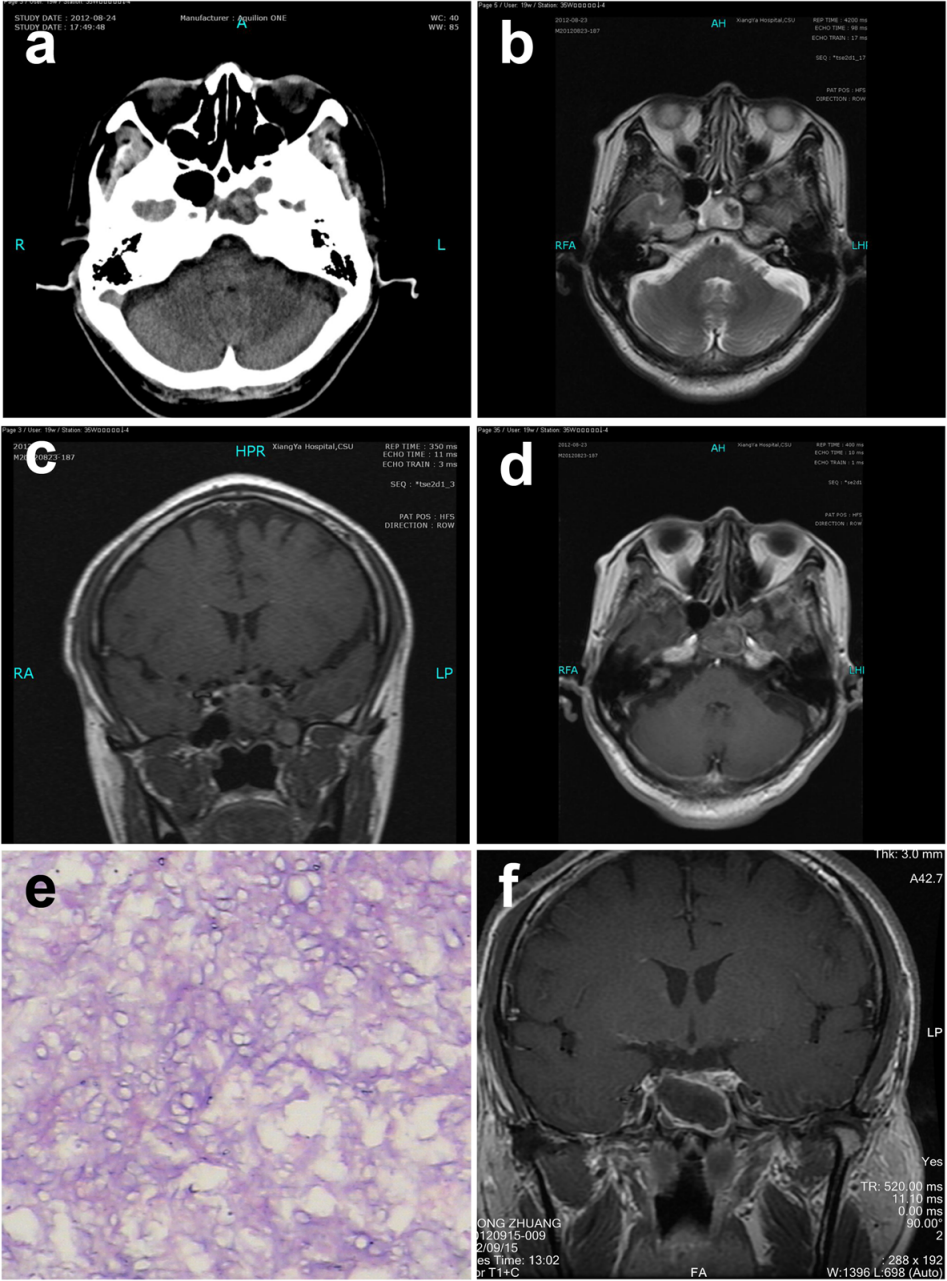
Case 2: a 52-year-old man. **a** CT image showing bone destruction in the sphenoid sinus and cavernous sinus region. **b** MRI showing an intrasellar mass with hyperintense signal extending bilaterally into the cavernous sinus on T2WI. **c** MRI showing an intrasellar mass with hyperintense signal on T1WI. **d** Enhanced MRI showing the lesions ring enhancement. **e** Photomicrograph of the surgical specimen revealing the histologic findings of septate, clustered aspergillus hyphae. **f** MRI showing the resection of the mass

A 52-year-old male was admitted with a paroxysmal headache in the right parietal region accompanied by visual disturbance in the right eye for over 2 months. Both symptoms mostly occurred in the morning and could be partially relieved with ibuprofen. He had a 2-year history of diabetes mellitus type 2 well controlled with metformin and repaglinide. He was afebrile and neurological examination was unremarkable. CT scan showed a mass in sphenoid sinus and cavernous sinus with bone destruction (Fig. 2a). MRI showed a 20 × 25 mm circular intrasellar mass with hyperintensity T1WI and T2WI, heterogeneously gadolinium-enhancement, and adjacent meningeal enhancement. The lesion extended bilaterally into the cavernous sinus and formed a 12 × 11 mm mass (Fig. 2b–d). The patient underwent a trans-sphenoidal mass excision and debridement. The lesion was solid and cystic with yellow-brown fluid and a gray wax-like solid component. A biopsy was performed immediately and histopathologic examination revealed *Aspergillus* species (Fig. 2e). Post-operative physical and neurological examination showed obvious improvement of headache and visual disturbance. Post-operative images indicated the resection of the mass (Fig. 2f). Examination after a follow-up of 20 months showed no clinical symptoms.

### Case 3

**Fig. 3:**
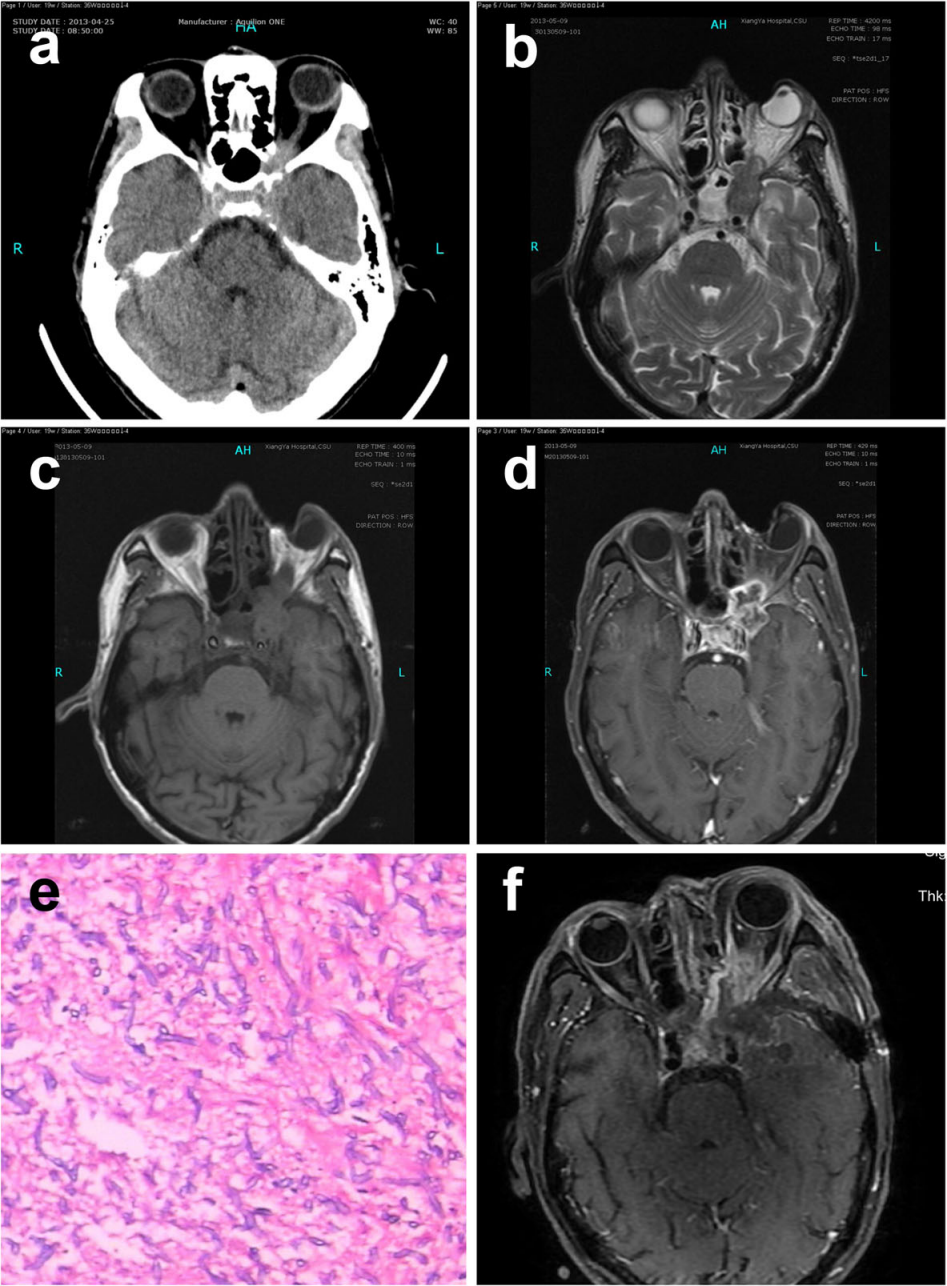
Case 3: a 64-year-old man. **a** CT image showing bone destruction in the left wall of the sphenoid sinus. **b** MRI T2WI showing 33 × 16 mm hypo-isointense lesion in both on the left optic canal region and extending into the orbit. **c** MRI T1WI showing an isointense signal of the lesion. **d** Enhanced MRI showing ring enhancement of the lesion. **e** Photomicrograph of the surgical specimen revealing the histologic findings of the septate, clustered aspergillus hyphae. **f** MRI showing the resection of the mass

A 64-year-old male had experienced a paroxysmal pain in the frontal region and left nose radiating to the occipital region for 7 months. The patient also reported progressive paresthesia on the left cheek and rapidly decreasing visual acuity in the left eye. Neurological examination revealed a left-sided ptosis as the only new finding. Interestingly, body temperature (axillary) fluctuated between 36.1 °C and 38.4 °C. The patient denied a history of other diseases. Physical examination showed no light perception or reflex was detected in the left eye. His pupils were 3 mm on the right and 4 mm on the left. CT showed an isodense lesion in the region of the left cavernous sinus, left orbital apex, left optic nerve canal, and sphenoid sinus. Bone destruction was seen in the left wall of the sphenoid sinus (Fig. 3a). MRI showed a 33 × 16 mm isointense lesion in both T1WI and T2WI of the left optic canal region and extending into the orbit. Enhanced-MRI showed ring enhancement of the lesion. Lesions with hyperintense T2-weighted signal were seen in paranasal sinuses (Fig. 3b–d). The patient was self-treated with anti-infectious medication but his headache increased in intensity, frequency, and duration. The patient underwent mass excision of the mass via pterional craniotomy. Cream-like liquid amidst a gray growth was observed in the left cavernous sinus. A biopsy was performed immediately, and histopathologic examination revealed an *Aspergillus* species (Fig. 3e). Post-operative images indicated the resection of mass (Fig. 3f). The patient received voriconazole after surgery. However, he was lost to follow-up.

### Case 4

**Fig. 4:**
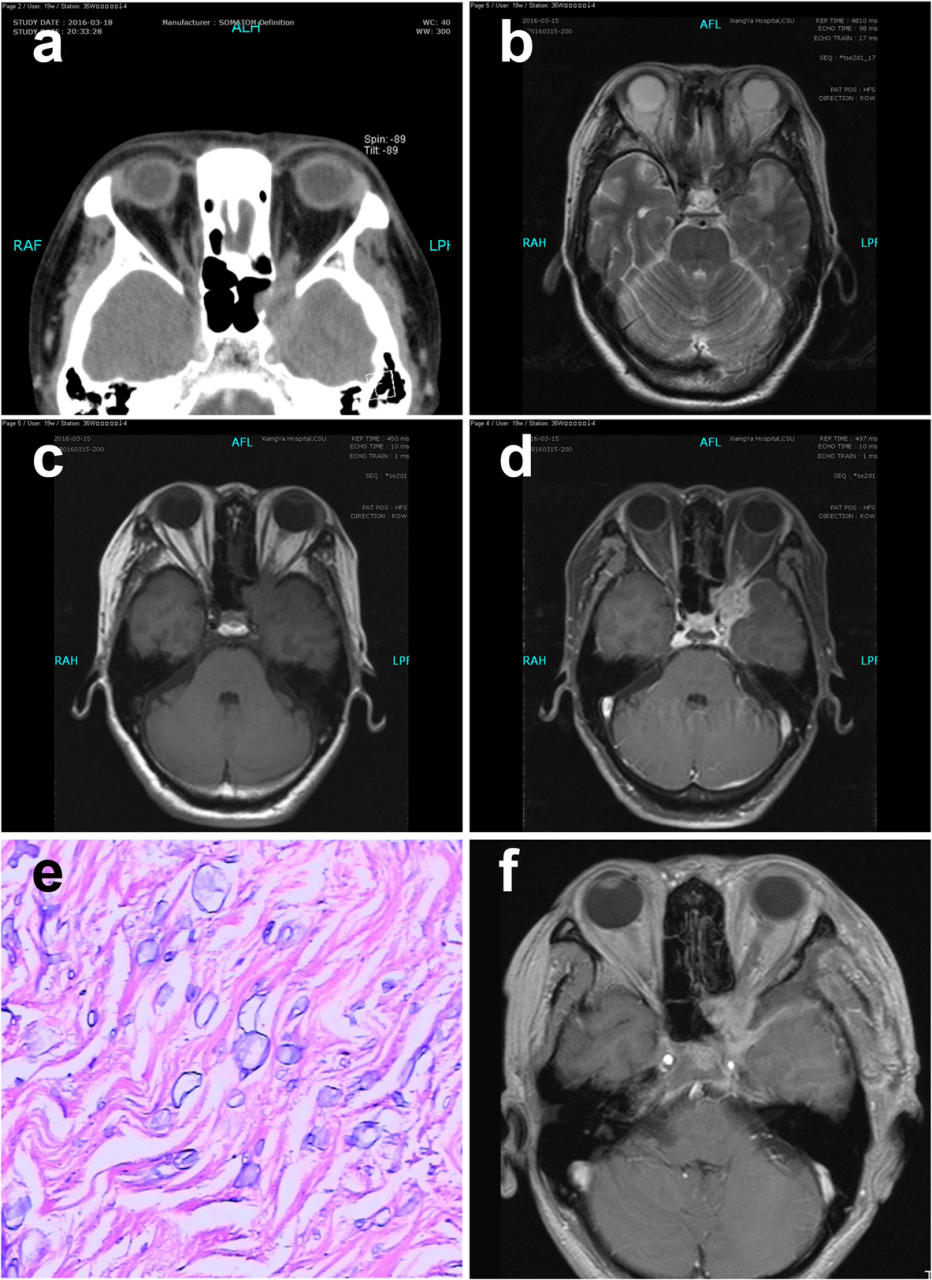
Case 4: a 50-year-old woman. **a** CT image showing an isodense lesion within the region of the left cavernous sinus left orbital apex, left optic nerve canal, and sphenoid sinus, with bone destruction in the left wall of the sphenoid sinus. **b** MRI showing a patchy lesion with the hyperintense signal on T2WI in the left cavernous sinus region. **c** MRI T1WI showing an isointense signal of the lesion. **d** Enhanced MRI showing noticeable enhancement of the lesion. **e** Photomicrograph of the surgical specimen revealing the histologic findings of septate, clustered aspergillus hyphae. **f** MRI showing the resection of the mass

A 50-year-old female was admitted with a paroxysmal boring pain in the left parietal and left frontal region for 9 months, accompanied by progressive vomiting and decreasing of visual acuity and proptosis in the left eye. Headache could be relieved by diclofenac sodium. Two months prior to presentation, her headache worsened extending to the whole left side of the face, accompanied by numbness. No light perception or reflex was detected in the left eye. Horizontal movement was limited in the left eye. Pre-operative CT showed a 29 × 20 mm isodense lesion with heterogeneously enhancement in the region of the left cavernous sinus, left orbital apex, left optic nerve canal, and sphenoid sinus. Bone destruction could be seen in the left wall of the sphenoid sinus. The optic nerve could not be distinguished from the lesion (Fig. 4a). MRI showed a 21 × 26 mm patchy lesion that was isointense on T1WI and hyperintense on T2WI in the left cavernous sinus region (Fig. 4b, c). Enhanced-MRI showed noticeable enhancement of the lesion. The lesion was closely related to the dura mater and extended to the sphenoid sinus and optic apex, partly surrounding the left internal carotid artery (Fig. 4d). MRA suggested stenosis in the cavernous segment of the left internal carotid artery and A1 segment of the left anterior cerebral artery. The patient underwent partial mass excision via a pterional craniotomy due to its close attachment to the ophthalmic branch and maxillary branch of the trigeminal nerve. The lesion was partly fibrotic and partly a milk-white viscous substance. A biopsy was performed immediately and histopathologic examination revealed *Aspergillus* species (Fig. 4e). The patient was administered voriconazole after surgery. Post-operative images indicated the resection of the mass (Fig. 4f). Examination after a follow-up of 15 months showed a complete resolution of the headache. Her vision of the left eye was not regained.

### Systematic literature review

A total of 42 publications were extracted from the literature for analysis. In those studies, 68 patients were found with pathologically confirmed *Aspergillus fumigatus*. The median number of patients in each study was 1 (range, 1–6). See Table 1 for a list of included studies and patient demographics. The mean patient age was 59 (range 8–82) years, and 38 (58.5%) patients were male. Thirty (44.1%) patients were found to be immunocompromised.

**Table 1:** Systematic literature review of cavernous sinus syndrome caused by *Aspergillus*

	Author	Year	Number	Age	Male
1	Kumar [6]	2017	8	33.8	5
2	Rosenvald [7]	2016	1	55	0
3	Wang [8]	2017	5	40.2	1
4	Brenet [9]	2016	1	75	1
5	Neil [10]	2016	1	69	1
6	Chi [11]	2014	1	55	1
7	Singh [12]	2014	1	68	1
8	Horowitz [13]	2013	1	57	1
9	Chan [14]	2012	1	64	1
10	McClelland [15]	2012	1	60	1
11	Lee [16]	2012	1	73	1
12	Furtado [17]	2011	1	30	1
13	Takahashi [5]	2011	4	67.8	2
14	Yan [18]	2011	1	56	1
15	Al-radadi [19]	2011	1	35	0
16	Saini [20]	2010	6	45	2
17	Wipfler [21]	2009	1	68	1
18	Cheung [22]	2009	1	49	1
19	Chua [23]	2008	2	41	1
20	Sasindran [24]	2008	1	8	1
21	Akhaddar [25]	2007	1	62	1
22	Freudenstein [26]	2007	1	66	1
23	Baumann [27]	2007	3	/	/
24	Stodulski [28]	2006	1	65	1
25	Browning [4]	2006	1	83	0
26	Chopra [29]	2006	1	66	0
27	Pinzer [30]	2006	1	59	0
28	Siraj [31]	2005	1	62	1
29	Deveze [32]	2005	2	62	0
30	Urculo [33]	2005	1	65	0
31	Petrick [34]	2003	1	74	1
32	Safdar [35]	2002	1	68	0
33	Endo [1]	2001	1	55	1
34	Hurst [36]	2001	1	73	1
35	Chandra [37]	2000	3	38.7	0
36	Imai [38]	1999	1	47	1
37	Takahashi [39]	1998	1	78	1
38	Carta [40]	1998	1	73	1
39	deShazo [41]	1997	2	61.5	1
40	Breadmore [42]	1994	1	71	1
41	Fujiwara [43]	1989	1	60	1
42	Rowed [44]	1985	1	82	1

The most common symptom was headache (*n* = 68, 61.8%), followed by vision impairment/loss (57.4%), ophthalmoplegia (54.4%), facial/orbital pain (30.9%), and exophthalmos (30.0%). The complete listing of clinical presentations was summarized in Table 2.

**Table 2:** Clinical findings of 68 patients

Headache	42 (61.8%)
Vision impairment/loss	39 (57.4%)
Ophthalmoplegia	37 (54.4%)
Facial/orbital pain	21 (30.9%)
Exophthalmos	19 (30.0%)
Diplopia	11 (26.2%)
Ptosis	11 (26.2%)
Altered sensorium	9 (13.2%)
Hemiparesis	4 (5.9%)
Weakness	3 (4.4%)
Mydriasis	3 (4.4%)
Rhinorrhea	1 (1.5%)
Eyelid drooped	1 (1.5%)
Horner	2 (1.5%)
Orbital apex involved	28 (41.2%)

The radiologic findings were found in some of the papers. Forty-seven cases included MRI results (T1WI *n* = 47, T2WI *n* = 42) and 41 cases included CT results. Among all patients, hypointense T1-weighted and hypointense T2-weighted were most common. Over half (*n* = 47, 53.2%) of the cases found an enhancement in T1WI. Bone destruction was observed on CT in 32 (78.0%) patients. Fifty-five cases out of 68 demonstrated treatment and prognosis. Surgery was performed in 37 (67.3%) patients and anti-fungal drugs were administrated in 48 (87.3%) patients. Amphotericin B (*n* = 55, 40%) and voriconazole (40%) were the most popular choice for antifungals. Despite the treatment, only 27 (50%) patients achieved complete recovery and 17 patients (31.5%) died from the disease.

## Discussion

In the pre-antibiotic era, the mortality rate of acute fulminant invasive fungal sinusitis was 50–80% [2]. Invasive fungal sphenoid sinusitis is more aggressive than invasive fungal infection of the other paranasal sinuses due to the involvement of important surrounding structures (e.g., cavernous sinus, pituitary gland, internal carotid artery, and cranial nerves II, III, IV, V1, V2, and VI). Clinical manifestations of significance include superior orbital fissure syndrome, orbital apex syndrome and cavernous sinus syndrome [45, 46]. When the infection spreads to the cavernous sinus, cranial neurologic and ophthalmologic symptoms may occur. Patients are usually asymptomatic during the early stages of the disease but are frequently misdiagnosed as a neoplasm or sellar abscess due to the lack of specificity on radiological images when it progresses. Takahashi et al. have found plasma ß-D-glucan levels are one of the most sensitive markers of deep fungal infection in patients with intracranial aspergillosis. However, these findings were not verified by other studies [5]. The low prevalence, atypical modes of onset and presentation, and low positive rate of *Aspergillus* in blood and CSF cultures also contribute to the difficulty of diagnosis [8]. Early diagnosis and proper intervention are crucial for patients to achieve a better prognosis [3].

### Presentation and physical examination

The early clinical symptoms of acute fulminant invasive fungal sinusitis include nasal obstruction, rhinorrhea, facial pain, headache, proptosis, and diplopia. In our cases, all four patients developed a severe headache and visual disturbance. One patient also had exophthalmos and another experienced altered sensorium. In accordance with previous literature, headache (61.8%) and visual disturbance (57.4%) were indeed the most common symptoms seen in patients with invasive sphenoid sinus aspergillosis. Fever is commonly not present. However, abnormal body temperature along with immunocompromised status is highly suggestive.

### Imaging characteristics

Imaging is widely used in neurological diseases but found to be limited in differentiating intracranial aspergillosis from meningiomas, tuberculomas, lymphoid malignancies, and metastasis [20]. Together with our cases, 52.9% (*n* = 51) of the patients presented enhancement on T1WI. 68.3% (*n* = 41) of the patients presented with hypo-iso signal intensity on T2WI. For computed tomography imaging, 86.7% (*n* = 45) were found to have bone destruction. Besides that, soft tissue with enhancement, sclerosis, and calcification of the mass were also found in some patients. On CT images, the fungal mass often appears hyperdense due to calcium salts and metal ions in necrotic areas [47]. Despite the lack of specificity, Kumar et al. concluded that CNS aspergilloma manifests differently in immunocompromised and immunocompetent patients. Reactive resorption to permeative destruction is mostly found in immunocompetent patients. While the mass is often presented as ring-enhancing lesions in immunocompromised patients, it is often presented as focal and thick pachymeningeal enhancement in immunocompetent patients [6]. In conclusion, certain imaging findings such as low signal intensity on T2W images, bone destruction on CT, and the presence of sinus disease with features of fungal infection could be suggestive. In some cases, imaging features like a heterogeneous parenchymal lesion with areas of hemorrhage, infarction, and vascular narrowing or obstruction can also be useful for diagnosis [20].

In addition to conventional imaging studies, DWI is exceptionally useful in differentiating ring-enhancing lesions due to brain abscess from neoplastic lesions [48]. There is a trend of characterizing the etiologic agents on the basis of DWI. Some investigations suggest that DWI appears to be the most sensitive modality for early identifications of cerebral aspergillosis [49–51] {Friedlander, 2003 #570}. Abscesses are usually hyperintense on DWI indicating restricted diffusion, while neoplastic lesions are hypointense or variable [52]. Furthermore, Luthra et al. found possible reliable indications suggesting fungal infection. They concluded that a ring-enhancing T2 heterointense lesion with irregular walls and irregular projections into the cavity with low ADC and no contrast enhancement of these projections carries a high probability of being a fungal abscess [53]. However, the fungal species still needs to be determined with histologic methods.

### Differential diagnosis

A CNS lesion with sinonasal involvement should raise suspicion towards an etiology of neoplasm, inflammation, and infection. Imaging provides limited value in differentiating among infections and a definite diagnosis must be made via biopsy. However, CNS aspergillosis has specific features that differentiate it from others, which are elaborated in this section.

Meningioma can present near the cerebral convexity in the parasagittal region, sphenoid wing, and the juxta sellar area. Rarely, it can arise in the orbit and paranasal sinuses, further extend into cavernous sinus [54]. Both aspergilloma and meningioma appear hyperdense and lead to lytic bone destruction. However, meningioma appears isointense on both T1WI and T2WI. In contrast, aspergilloma appears hypointense on T2WI [55].

Trigeminal schwannoma can also occur in the cavernous sinus and exert a mass effect on adjacent structures. It sometimes contains cystic areas causing heterogeneously enhancement on both MRI and CT, which make it difficult to be distinguished from aspergillosis. However, it is typically hyperintense on T2WI and restricted diffusion is usually not found on DWI [56].

Pituitary adenomas invading the cavernous sinus are rare lesions which can be confused with Aspergilloma clinical wise [57]. The encasement of the ICA is also seen. The mass is typically isotense on T2WI but can be heterogenous and vary in signal due to areas of cystic change, necrosis, or hemorrhage [58, 59]. DWI may be applied to assist differentiation as discussed above. Sphenoid sinus ectopic pituitary adenoma is another extremely rare condition. It is usually heterogeneous, demonstrating foci of hypointensity on T1WI and hyperintensity on T2WI [60].

Inflammation of orbital, paranasal sinuses and brain are hypointense on T2WI and may show marked post-contrast enhancement. Bony erosions and destruction are also common in inflammatory diseases [61]. Wegener granulomatosis can mimic and show hypointense T2WI and contrast enhancement on MRI [62]. However, Wegener granulomatosis is a systemic vasculitis. The majority of the patients will have concurrent pulmonary, renal, and skin involvement. Additionally, inflammatory markers and c-ANCA can further exclude the disease.

### Treatment and outcomes

Surgery is the only management treatment method common to all previous publications that significantly change patients’ outcomes. Surgical procedure includes the triple aim of diagnosis, cure, and prevention of a recurrence [9]. Decompression of the optic nerve is applied when optic canal is involved. Surgical treatment should also aim at radically removing the infectious mass rather than draining alone [30]. When combined with previous literature, our four cases demonstrate the importance of surgery. 44.4% (*n* = 18) of the patients without surgery eventually died, while only 24.4% (*n* = 41) of the patients with surgical treatment died from the disease. However, in immunocompromised patients, the risk of operation should be taken into consideration. Anti-fungal usage is also recommended by most studies. Clinically, voriconazole is considered the drug of choice for invasive aspergillosis [25]. Amphotericin and voriconazole are the most widely used; however, there is no relation between drug preference and prognosis. Fungal cavernous sinus infection may further cause a secondary bacterial infection thus anti-bacterial is always applied for precautionary purposes. Cavernous sinus infection is known to possibly lead to inflammatory reaction and hypercoagulability. Whereas, anticoagulation is still controversial [63, 64]. Current recommendations call for initial intravenous heparin and at least 6 months’ treatment of warfarin. The optic nerve is found with the poorest recovery rate.

## Conclusion

Invasive sphenoid sinus aspergillosis is a rare yet lethal condition that commonly presents with headache and visual disturbance. Surgery might not be timely when clinicians misdiagnose it due to its sellar tumor-like character and can cause grave consequences. Here, we meant to gather reported cases to determine specific findings that assist in accurate diagnosis and proper treatment. At least 68 reported cases exist in the English literature, which yielded 72 analyzable cases together with our patients. Overall, both lab tests and radiologic image findings were not specific. However, enhancing T1WI, hypointense T2WI, and boney destruction on CT is highly suggestive of this diagnosis. The involvement of the optic canal and cavernous sinus should raise concern. In addition, DWI could also be considered and low ADC may allow early diagnosis and treatment. ß-D-Glucan levels are also found to be one of the most sensitive markers in some patients with intracranial aspergillosis. Although the function of most intracranial nerves could be regained, the optic nerve was found to have the poorest recovery rate. We recommend prompt complete surgical resection, including curettage, once diagnosed to achieve a better clinical benefit. Anti-fungal agent use should also be considered for precautionary purposes or if complete resection cannot be achieved.

## Data Availability

Data sharing is not applicable to this article as no datasets were generated or analyzed during the current study.

## Abbreviations

ADC: Apparent diffusion coefficient
CNS: Central nervous system
CT: Computed tomography
DWI: Diffusion-weighted imaging
MRI: Magnetic resonance imaging
T1WI: T1-weighted imaging
T2WI: T2-weighted imaging
